# Central Hypoventilation: A Rare Complication of Wallenberg Syndrome

**DOI:** 10.1155/2018/4894820

**Published:** 2018-03-21

**Authors:** Keithan Sivakumar, Manveer Garcha, Dev Mehta, Megan C. Leary, Hussam A. Yacoub

**Affiliations:** ^1^Lehigh Valley Physician Group-Neurology, Lehigh Valley Health Network, Allentown, PA, USA; ^2^Morsani College of Medicine, University of South Florida, Tampa, FL, USA

## Abstract

Central alveolar hypoventilation disorders denote conditions resulting from underlying neurologic disorders affecting the sensors, the central controller, or the integration of those signals leading to insufficient ventilation and reduction in partial pressures of oxygen. We report a patient who presented with a left lateral medullary ischemic stroke after aneurysm repair who subsequently developed a rare complication of CAH. Increased awareness of this condition's clinical manifestations is crucial to make an accurate diagnosis and understand its complications and prognosis.

## 1. Introduction

Central alveolar hypoventilation (CAH) disorders arise from improper signal integration of the central respiratory drive center in the lower brainstem, which can be due to congenital or acquired neurologic dysfunction. These disorders can lead to hypercarbia, hypoxia, and insufficient ventilation. The disorder typically presents in sleep alone but in severe cases can present during sleep and arousal states.

Acquired central hypoventilation typically occurs due to traumatic, ischemic, or inflammatory injuries to the brainstem. The term “Ondine's curse” was first used by Severinhaus and Mitchell [1] to describe patients who were able to breathe voluntarily after undergoing bilateral spinothalamic tract cordotomies but subsequently became apneic during sleep. This loss of reflex breathing was determined to be due to poor ventilatory responsiveness to inhaled carbon dioxide. Unilateral lesions rarely cause hypoventilation.

Brainstem infarctions and ischemia can lead to CAH [2–4]. Central hypoventilation and apnea can occur secondary to watershed infarcts in the fetal and neonatal tegmentum [4]. Brainstem tumors including gliomas and acoustic neuromas can lead to central hypoventilation [5, 6]. Brainstem tumors in children often manifest with sleepiness due to compression [7], which can improve with resection of the tumor [5]. Paraneoplastic and viral encephalitis as well as bulbar polio can result in central hypoventilation. Ventilatory management is typically supportive and outcome is variable and unpredictable [8–11].

## 2. Case Presentation

A 62-year-old woman with no significant medical history experienced an acute onset of vertigo after abruptly turning her head to the left. The symptoms resolved within 24 hours. Computed tomography (CT) of the head and CT angiography of the head and neck revealed an unruptured 11.7-millimeter wide-necked left posterior inferior cerebellar artery (PICA) aneurysm that was subsequently confirmed with conventional cerebral angiography. She was taken to the operating room where she subsequently had the aneurysm clipped. Intraoperatively, the patient developed profound bleeding due to loss of local hemostasis. As a result, the patient had an episode of pulseless electrical activity, followed by resuscitation and consequent sacrifice of the left vertebral artery.

Postoperatively, the patient remained intubated due to several episodes of prolonged apnea mainly during sleep, having often to be “reminded” to breathe. Initial physical examination was significant for left eye ptosis and meiosis, subtle right gaze preference, and left-sided weakness and ataxia. Noncontrast magnetic resonance imaging of the brain demonstrated increased T1 signal in the left inferior cerebellum and medulla in the PICA vascular distribution. It was noted that, during sleep when on the same ventilator settings as those during wakefulness, the patient became acidotic and was retaining carbon dioxide (CO_2_), with a partial pressure of CO_2_ (PaCO_2_) of 76 millimeters of mercury, while breathing only at the set rate and not initiating her own breaths. During wakefulness, the patient was able to maintain ventilation, evident by normal PaCO_2_. Over the course of stay in the intensive care unit, the patient was maintained on different modes of assisted ventilators and eventually continuous positive airway pressure. Consequently, the apneic episodes progressively became shorter until the patient recovered spontaneously before discharge to an acute rehabilitation facility.

## 3. Discussion

CAH syndrome, also known as “Ondine's curse,” is an exceedingly uncommon disorder categorized by failure of respiratory mechanisms while asleep [8]. Patients with this condition have no difficulties during wakefulness but have persistent apnea during sleep [9, 10].

Congenital CAH is very common in the neonatal period and has been reported in preterm infants. Neonatal CAH occurs in infants who suffer birth asphyxia or metabolic insults. The pathogenesis of CAH in the neonatal period is thought to be related to delayed synaptogenesis associated with genetic diseases or certain cerebral malformations [12]. Many have discussed certain congenital malformations or chromosomopathies involved in the congenital form of CAH, including a PHOX2B mutation first documented in 2009 by Lee et al. [13] or abnormal development of the neural crest as a result of the RET-GDNF signaling pathway [8, 14].

There are numerous etiologies of this pathological conundrum which include infectious, malignant, upper cervical cord injury, various degenerative diseases, demyelinating diseases, mitochondrial disease, and ischemia [15–18]. Furthermore, brainstem involvement in ischemic infarctions and dysregulation of the autonomic control of breathing have been infrequently described [18, 19]. Synaptic failure in the nucleus solitarius indeed can occur with metabolic or hypoxic/ischemic insults, followed by recovery of those neurons that were rendered nonfunctional for a transitory period without neuronal loss.

Among the various aberrant ventilatory patterns, CAH is the pattern that allows for the most accurate localization of the lesion, typically in the lower brainstem involving the lateral medulla. This lesion can cause selective interruption of the anterolateral medullocervical pathway, a descendent pathway normally responsible for autonomic breathing control [4].

In the case presented above, the patient had a lesion in the left lateral medulla, which produced many of the classic symptoms of Wallenberg syndrome: ipsilateral arm and leg ataxia, Horner's syndrome, facial weakness, and contralateral arm and leg sensory disturbance. The presumed central hypoventilation was related to compression and swelling of the medulla, affecting both the ventral and dorsal respiratory groups of the autonomic breathing center.

Laboratory criteria for the diagnosis of CAH are not well established [8–10, 20]. Some authors propose that prolonged and persistent periods of apnea associated with desaturation and hypercapnia during nonrapid eye movement sleep should be demonstrated for diagnosis. Other diagnostic criteria include (a) normal pulmonary and mediastinal anatomy, (b) normalization of partial pressure of oxygen through voluntary breathing when awake, and (c) precipitation of alveolar hypoventilation with diminished voluntary control (i.e., sleep). The clinical picture and location of the ischemic lesion in the case presented here support the diagnosis of CAH [21].

Both pharmacological and supportive treatments have been proposed for CAH. The goal is to activate the remaining respiratory nuclei through induction of metabolic acidosis. Pharmacological agents such as trazodone, acetazolamide, medroxyprogesterone, protriptyline, clomipramine, and caffeine have been used [22, 23]. Supportive measures include the use of a diaphragmatic pacemaker, with a reported success rate of 50–70% in some series [9].

In patients with acquired CAH, spontaneous recovery without the need of a pacemaker, as was the case in our patient, is frequent. These patients usually respond well to tracheostomy and nocturnal-assisted ventilation leading to spontaneous recovery [24]. Death related to CAH usually occurs during sleep, presumably due to complete apnea, which can be alleviated if adequately ventilated [24]. Prognosis is variable and depends on the specific location of the lesion, and recovery is usually unpredictable.

The term Ondine's curse is more commonly used to describe cases of congenital CAH but can be applied to acquired cases due to trauma, ischemia, or inflammation [18]. The patient in the case discussed here developed CAH secondary to bleeding complications during aneurysm repair. Unlike other reported cases, CAH in our patient was related to compression and swelling of the medulla, subsequently affecting both the ventral (pre-Bötzinger nucleus and vagus nucleus ambiguus) and dorsal (nucleus tractus solitarius) respiratory groups of the autonomic breathing center [1].

This case extends our knowledge of CAH and increases awareness of its etiologies, diagnosis, and complications.
